# Prenatal diagnosis and implications of microphthalmia and anophthalmia with a review of current ultrasound guidelines: two case reports

**DOI:** 10.1186/s13256-018-1746-4

**Published:** 2018-08-29

**Authors:** A. Searle, P. Shetty, S. J. Melov, T. I. Alahakoon

**Affiliations:** 1 Westmead Institute for Maternal and Fetal Medicine, Westmead Hospital, Corner Hawkesbury Road and Darcy Road, Westmead, NSW 2145 Australia; 2 The University of Sydney Westmead Clinical School, Sydney, NSW Australia

**Keywords:** Microphthalmia, Microphthalmos, Anophthalmia, Anophthalmos, Guidelines, Ultrasound

## Abstract

**Background:**

Microphthalmia and anophthalmia are rare congenital fetal abnormalities. The combined incidence is estimated at 1 in 10,000 births. These two conditions arise from complex and incompletely understood genetic and/or environmental causes. Prenatal diagnosis is neither frequent nor easy and relies on precise, high-quality ultrasonography. Current antenatal ultrasound protocols for imaging of the fetal eye are inconsistent and inadequate to screen for the spectrum of ocular malformations, and there are no clear guidelines on detection of these rare abnormalities. Our study of two cases highlights the importance of early detection, and we review current practice and suggest a definitive fetal imaging protocol.

**Case presentation:**

We present two antenatal cases, one each of microphthalmia and anophthalmia, both diagnosed at the morphology scan at our tertiary fetal medicine unit. In both cases, the parents (a 36-year-old woman of Mauritanian ethnicity and a non-consanguineous partner of Nepalese descent, and a 31-year-old Caucasian woman and non-consanguineous Caucasian partner) elected to terminate their pregnancies and made unremarkable recoveries. Subsequent fetal autopsy confirmed the ultrasound scan findings.

**Conclusions:**

We recommend that antenatal ultrasound guidelines are updated to specify use of a curvilinear transducer (2–9 MHz) to image both orbits in the axial and coronal planes, aided by use of a transvaginal probe when the transabdominal approach is inadequate to generate these images. When applicable, three-dimensional reverse-face imaging should be obtained to aid the diagnosis. The presence, absence, or non-visualization of lenses and hyaloid arteries should be documented in reports and these cases referred for a tertiary-level ultrasound scan and fetal medicine review. Imaging of the orbits should occur from 12 weeks’ gestation. Magnetic resonance imaging and amniocentesis with chromosome microarray testing may provide additional genetic and structural information that may affect the overall morbidity associated with a diagnosis of microphthalmia or anophthalmia.

## Background

Microphthalmia and anophthalmia are rare congenital abnormalities, occurring on a spectrum of congenital ocular disorders including congenital cataracts, cryptophthalmos, cyclopia/synophthalmia, congenital cystic eye, and coloboma. Microphthalmia and anophthalmia have both genetic and non-genetic causes, may be unilateral or bilateral, and may be found in isolation or as one component of a syndrome.

There is a general paucity of data on the prevalence of the conditions, and a lack of consistent, widely applied ultrasound guidelines for prenatal diagnosis. We discuss the diagnosis and current international ultrasound protocols for imaging fetal ocular structures. We also discuss ultrasound techniques and resources required for definitive identification of the associated morphological abnormalities. A brief overview of relevant early fetal development and the complex etiology of this serious prenatal diagnosis provides further useful context for treating clinicians.

## Case presentation

We present two cases of fetal ocular abnormalities referred to our tertiary center with unilateral anophthalmia and microphthalmia.

### Patient A

**Fig. 1:**
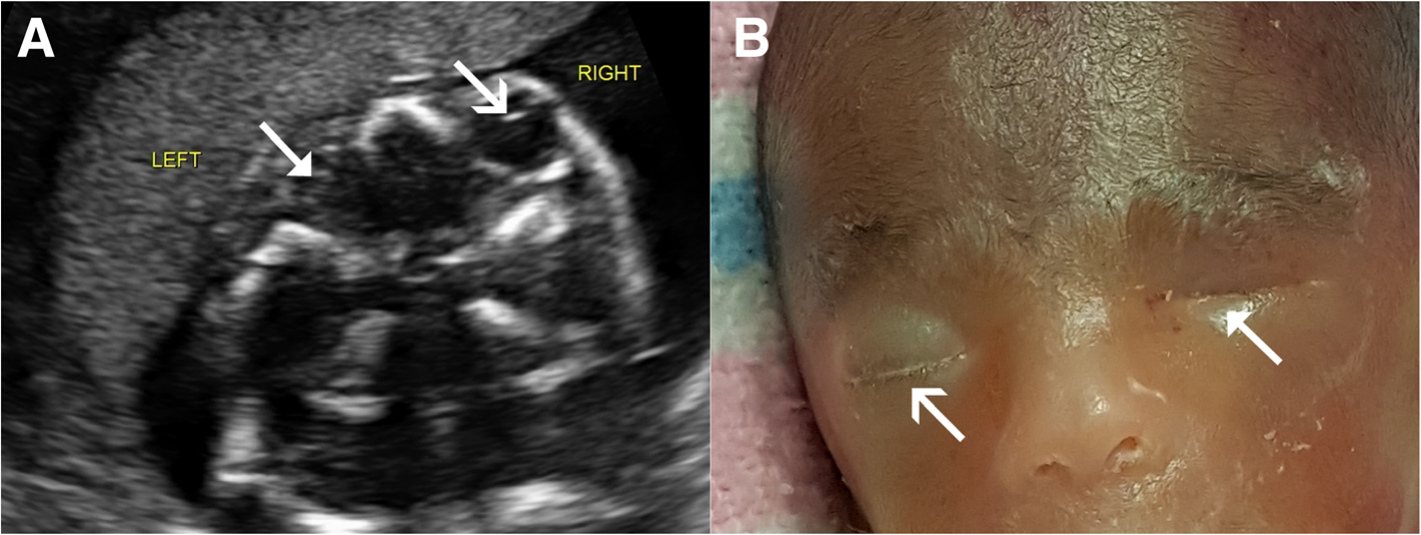
Case A. **a** Ultrasound findings of microphthalmia. **b** Postnatal view showing left-sided microphthalmia. *Triangle arrow-head* represents microphthalmia. The *simple wide arrow-head* represents the normally formed eye

Patient A was a 36-year-old woman of Mauritanian ethnicity who presented for an initial hospital-booking visit at 13 weeks’ gestation. She had no known medical conditions and a non-consanguineous partner of Nepalese descent. Her obstetric history included a 35-week morphologically normal stillbirth of unknown etiology. The pregnancy with which she presented had a low-risk result for the first-trimester aneuploidy antenatal screening in the nuchal translucency program. A fetal morphology scan attended at 19 weeks identified potential fetal anomalies, leading to a tertiary referral for review. A detailed sonogram at 21 weeks’ gestation confirmed left microphthalmia (Fig. 1a) and a small biparietal diameter (< fifth centile).

A range of investigations and management options were offered and consented to, including: genetic counseling, amniocentesis, single nucleotide polymorphisms (SNP) array testing, placental histopathological testing, preservation of cell line, and a full postmortem. A magnetic resonance imaging (MRI) examination was declined by the parents. Amniocentesis and chromosomal microarray showed a chromosomally normal male and genetic counseling was organized. The couple had significant concerns regarding the uncertain prognosis, leading to a decision for an elective termination. The fetal postmortem showed left-sided microphthalmia (Fig. 1b), with associated persistent hyperplastic primary vitreous, probable hypoplasia to the left side of the face, and a thin left optic nerve compared to the right. Placental histopathological results were normal.

### Patient B

**Fig. 2:**
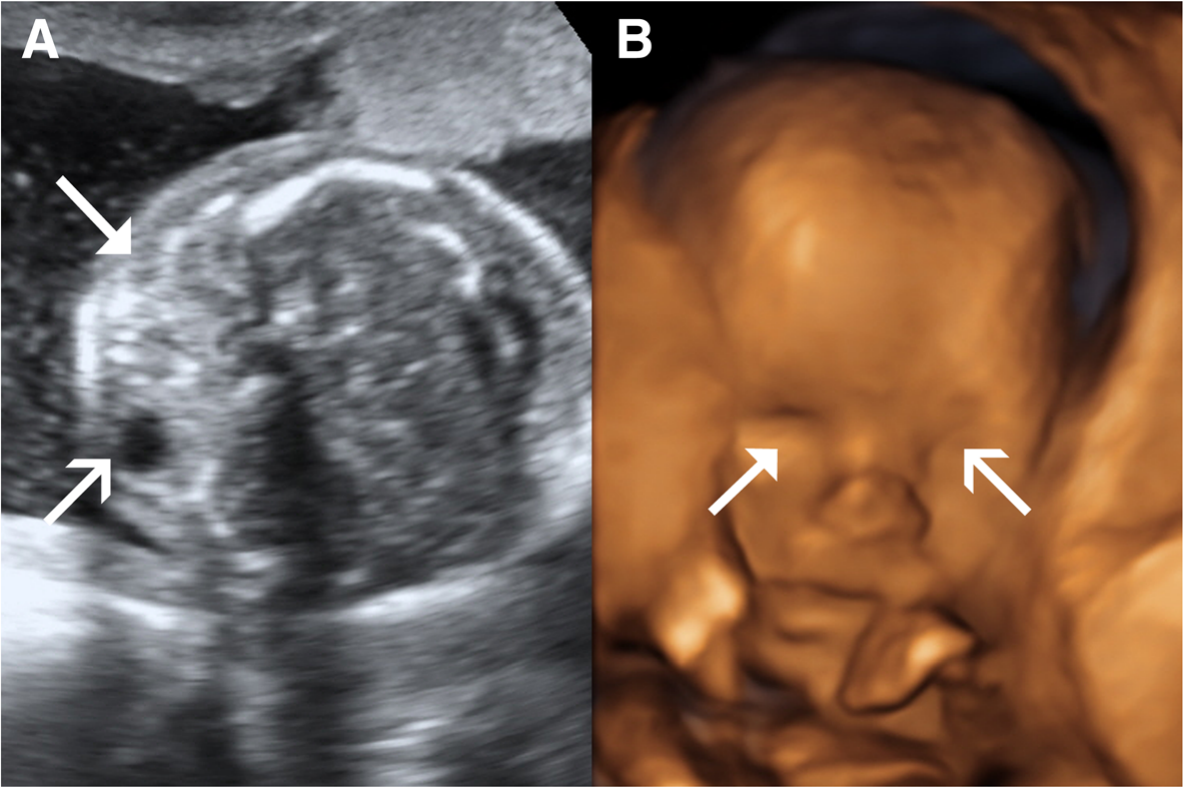
Case B. **a** Two-dimensional ultrasound findings of anophthalmia. **b** Three-dimensional ultrasound view of the face showing unilateral anophthalmia. The *triangular arrow-head* represents anophthalmia. The *simple wide arrow-head* represents the normally formed eye

**Fig. 3:**
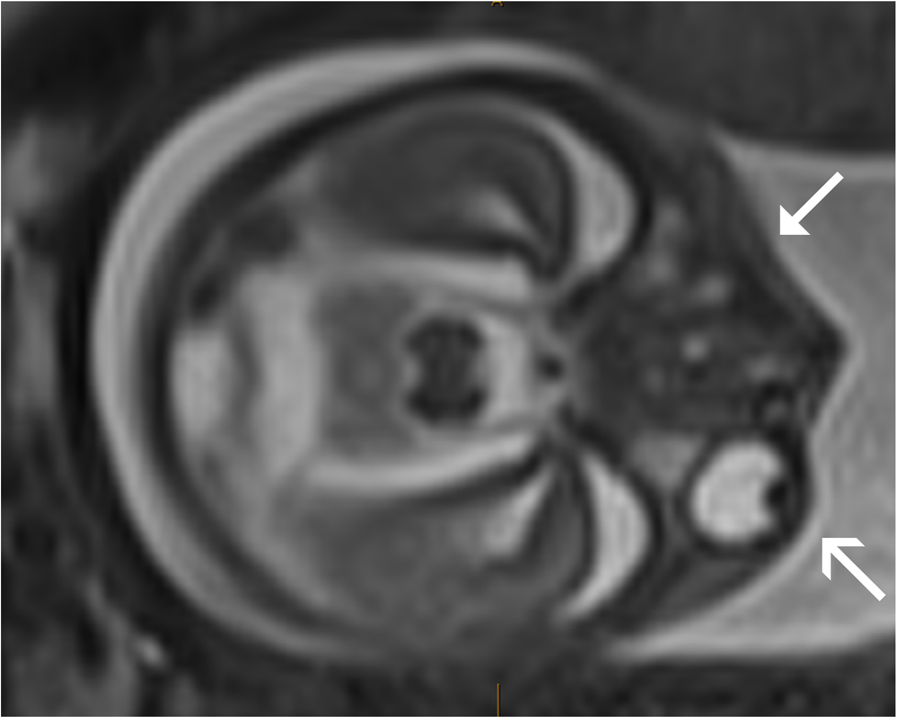
Magnetic resonance images of the fetus with anophthalmia, showing axial views of the fetal head at the level of the eyes. The *triangular arrow-head* represents anophthalmia. The *simple wide arrow-head* represents the normally formed eye

**Fig. 4:**
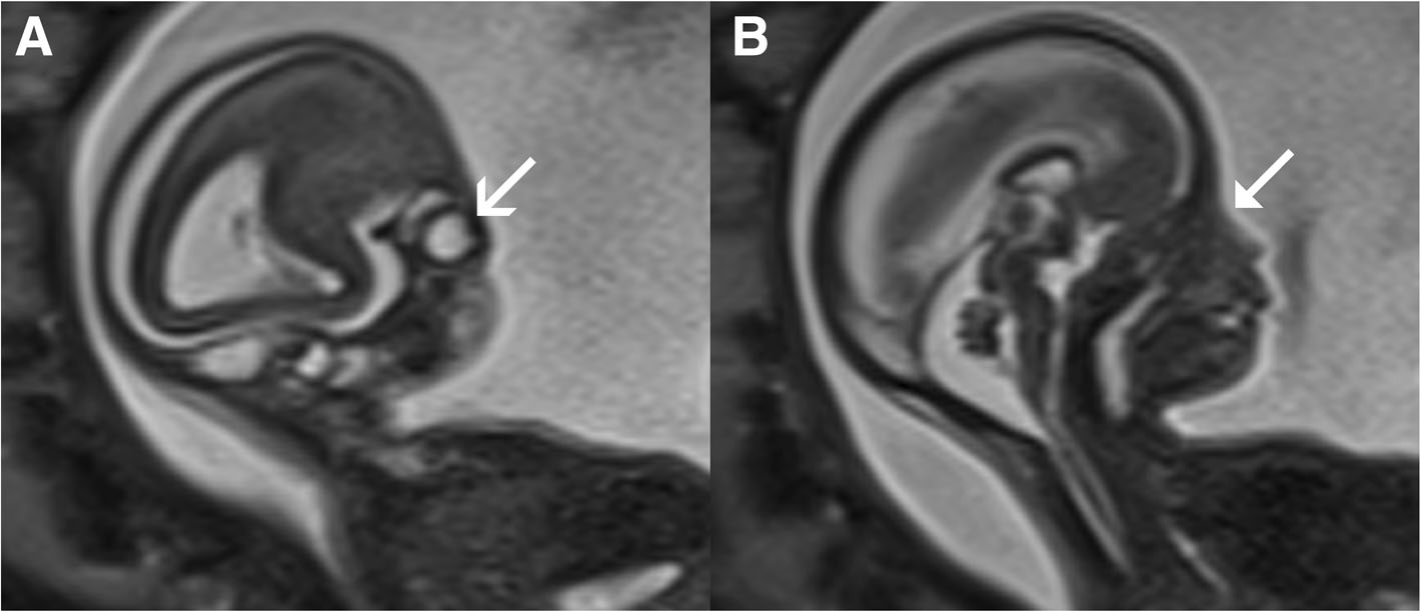
Magnetic resonance images of the fetus with anophthalmia, showing parasagittal views of the fetal head. **a** The normal eye; **b** anophthalmia. The *triangular arrow-head* represents anophthalmia. The *simple wide arrow-head* represents the normally formed eye

Patient B was a 31-year-old Caucasian woman with a non-consanguineous Caucasian partner and a history of a term normal birth followed by a first trimester miscarriage. She had no significant medical or family history and stated no illicit substance use. She presented with an uncomplicated pregnancy with a low-risk screening result on nuchal translucency for aneuploidy. At the 20-week fetal anomaly morphology scan, an absent right globe was identified (Fig. 2) with mild bilateral ventriculomegaly. Fetal MRI at 20 weeks further delineated the absent right globe, dysplastic ventricular system (Figs. 3 and 4), and confirmed diagnosis.

A screen for toxoplasmosis, rubella, cytomegalovirus, herpes simplex virus, listeria, parvovirus, and human immunodeficiency virus (HIV) (TORCH screen) completed at the time of diagnosis was negative. Our patient had no family history of fetal anomalies. Amniocentesis and microarray results showed no chromosomal anomalies in a male fetus. Our patient chose not to continue the pregnancy and a termination was performed without complication. An autopsy revealed right-sided anophthalmia with right optic nerve atrophy and mild bilateral ventriculomegaly. Placental histopathological results were normal. Genetic counseling and testing was organized.

### Methods

In both the cases, a Voluson E10 scanning machine was used (General Electric Medical System, Zipf, Austria) equipped with a convex 2–9 MHz, and a three-dimensional convex, 2–6 MHz volumetric transducer. In case A, we produced the images in two-dimensional ultrasound. In case B, both two-dimensional and three-dimensional ultrasound techniques were used, including the three-dimensional reverse-face technique. Images were obtained at the level of the orbits, from cranial to caudal end in axial as well as coronal planes. Orbits were examined for shape, intra-orbital and bi-orbital distance, as well as presence of lenses and hyaloid arteries. The fetal face was also examined in sagittal, axial, and coronal planes for any associated abnormalities.

## Discussion

Anophthalmia refers to complete absence of the globe in the presence of ocular adnexa (eyelids, conjunctiva, and lachrymal apparatus), and microphthalmia is defined as a globe with a total axial length that is at least two standard deviations below the mean for age [1]. Microphthalmia can be classified according to the anatomic appearance and severity of the reduction of the globe: severe microphthalmia refers to a globe with a corneal diameter less than 4 mm and a total axial length less than 10 mm at birth [2]. These ocular malformations can be unilateral or bilateral, and can be isolated or occur with other malformations as part of a syndrome. A normal ultrasound is not a guarantee that vision will be intact in an eye that appears normal.

The reported incidence of microphthalmia and anophthalmia varies in the literature and reflects a general paucity of reliable epidemiological data about this rare congenital abnormality. Based on hospital admissions of live babies, UK statistics from 1999 to 2011 describe the incidence of congenital anophthalmia as 0.04–0.24 per 10,000, and of congenital microphthalmia as 1.00–1.08 per 10,000 [3]. The estimated incidence from a retrospective cohort study of the Danish national patient registry in Denmark from 1995 to 2012 of microphthalmia and anophthalmia was 1.2 per 10,000 live births. Extra-ocular abnormalities were observed in 32.1% of these cases [4]. These data have the limitation of not including the number of women with a prenatal diagnosis of fetal microphthalmia or anophthalmia who chose to terminate the pregnancy, so the real incidence is likely to be higher. The 1989–1997 data from the California birth defects monitoring program identified the prevalence of anophthalmia in live births and stillbirths as 0.18 per 10,000 and for bilateral microphthalmia as 0.22 per 10,000 [5]. Unilateral microphthalmia was excluded from the data. The available data suggest that microphthalmia and anophthalmia are rare presentations, with upper estimates of incidence around 1 per 10,000 births.

As a spectrum of disorders, the etiology of anophthalmia and microphthalmia is complex and relates to the precise stage of embryological development affected by infection and/or gene mutation. Eye development commences around the third embryological week, with the development of two small grooves on the forebrain (optic grooves or sulci). Lengthening from the forebrain to the surface ectoderm, these optic grooves become the optic vesicles. The optic vesicles continue to grow, with narrowing of the proximal tissue connected to the forebrain forming the optic stalk and finally the optic nerve. The origin for the lens, the lens placode, is formed in the fifth and sixth week of gestation, and becomes complete by week eight. The double-layered optic cup is formed in weeks five and six, by an invagination of the optic vesicle to ultimately form the iris, ciliary body, and retina. The mesenchyme on the external surface of the optic cup forms the choroid and sclera in week six or seven [6, 7].

The pathogenesis of microphthalmia and anophthalmia is heterogenous and incompletely understood. Environmental agents have been implicated, with genetic factors and vascular disruption [1]. Chromosomal abnormalities (aneuploidy, deletions, rearrangements, single-gene disorders) are responsible for an unknown proportion of the presentations, and may be inherited or isolated. Single-gene deletions and mutations affecting the ability to produce proteins and enzymes such as *SOX2*, *MFRP*, *ALDH1A3*, *STRA6*, *PAX2*, and *OTX2*, have been identified as causing isolated microphthalmia and anophthalmia and syndromic forms [8–13]. *SOX2* and *PAX6* mutations may cause lens induction failure, *FOXE3* mutations are associated with lens agenesis, and *OTX2*, *CHX10*, and *RAX* may cause failure of retinal differentiation. All these mutations may cause organ dysgenesis, leading to microphthalmia or anophthalmia [1]. This list of causative genes is not comprehensive but highlights some of the commonly identified genes, and indicates the significant complexity of the genetics of anophthalmia and microphthalmia.

Syndromic forms are more likely to be associated with intellectual impairment. Known syndromes associated with anophthalmia and microphthalmia include X-linked recessive Lenz microphthalmia syndrome [14, 15], microphthalmia with linear skin defects syndrome [16], and anophthalmia–esophageal–genital syndrome [17]. Other authors implicate intra-uterine infection with TORCH organisms as causes for microphthalmia [7, 18]. The known environmental causes include alcohol, thalidomide, vitamin A deficiency, and hydantoin [1, 19, 20].

Patients with anophthalmia and microphthalmia experience a spectrum of visual impairment from mild impairment to complete blindness. The anomaly also presents cosmetic problems, which are treated variably with surgery and ocular prostheses [21]. As previously noted, patients may also suffer from intellectual disability, particularly if the anomaly presents as part of a syndrome. Prognosis is varied and patients usually require the support of a multidisciplinary team to manage the vision, plastic surgery, and intellectual and psychological aspects of the condition [1].

The use of ultrasound for diagnosis of fetal ocular defects was first described in 1991 [22]. The capacity for diagnosis has paralleled technical advancements in ultrasound. A diagnosis of anophthalmia can be made by two-dimensional ultrasonography when eyeballs and lens are absent. More information can now be gained with the advent of three-dimensional ultrasonography, especially when the fetal head position is unfavorable for two-dimensional ultrasound [2, 23]. The use of three-dimensional ultrasound is well known for prenatal diagnosis of fetal facial abnormalities [23]. In 2005, the ultrasound technique of three-dimensional reverse-face view was first described by Campbell *et al.* [24] for the diagnosis of cleft palate. Subsequently in 2012, Araujo *et al*. [25] described the reverse-face imaging technique to diagnose anophthalmia at a gestation of 30 weeks, with the advantage of not being affected by shadowing [2]. It is necessary to take a satisfactory three-dimensional volume, with adequate inclusion of both orbits [23]. The sagittal plane of the face of a fetus is rotated 180° in the *z*-axis, and the green line (region of interest) is placed at the level of fetal orbits. This results in the image of a three-dimensional reverse-face view. Other authors have noted the use of transvaginal ultrasound at approximately week 13 to assist in the early diagnosis of microphthalmia and anophthalmia in fetuses with parents or older siblings who have the condition [18].

Fetal MRI is an adjunct tool to ultrasound in diagnosing fetal anomalies, and can be particularly useful in identifying malformations of the central nervous system, providing detailed evaluation of the brain sulci, gyri, ventricles, cerebellum, corpus callosum, and other structures. MRI can confirm the absence of eye tissue, the optic nerve, and extra-ocular muscles, in cases of anophthalmia [25–27].

### International ultrasound protocols for identifying microphthalmia and anophthalmia

We searched international ultrasound protocols to review the current prenatal screening practices for microphthalmia and anophthalmia. We identified four commonly used protocols and suggested protocol guidelines in journal articles, which are summarized below.

#### International Society of Ultrasound in Obstetrics and Gynecology, 2010 guidelines [28]

These guidelines note the following in relation to imaging of the fetal face:

Minimum evaluation of the fetal face should include an attempt to visualize the upper lip for possible cleft lip anomaly. If technically feasible, other facial features that can be assessed include the median facial profile, orbits, nose and nostrils.

#### Fetal Medicine Foundation, 2002 guidelines [29]

These guidelines relate to the 18–23-week morphology scan, and note the need to image the orbits, specifying that the sagittal, transverse, and coronal planes are required. The coronal planes are described as the most important to view the orbits and face. A series of transverse scans from the top of the fetal head, moving caudally allows examination of various facial structures, including the orbits. These guidelines also discuss the orbit measurements and how these relate to the diagnosis of microphthalmia. The diagnosis depends on identifying a decreased intra-orbital diameter and then carefully identifying and defining the intra-orbital structures including lens, pupil, and optic nerve.

#### American Institute of Ultrasound in Medicine, 2013 guidelines [30]

These guidelines do not mention having a detailed examination of the orbits in the head, neck, and face category.

#### Australasian Society for Ultrasound in Medicine, 2014 guidelines [31]

The protocol for the 18–20-week obstetric scan was reviewed and now includes identification and measurement of the orbits. The protocol includes charts for the expected orbital measurements, by gestation. There is no specification on the depiction of lenses and the views needed to be obtained for optimal measurements.

### Protocol literature review

The published literature on ultrasound protocols for identification of microphthalmia and anophthalmia includes an original article on early sonographic detection of recurrent fetal eye anomalies. Mashiach *et al.* [18] described how transvaginal sonography at 14–16 weeks was helpful in detecting the eye abnormality in five cases in which at least one previous child in the family had the same congenital eye anomaly. The orbital region was best visualized using the coronal plane. These authors recommended offering a detailed targeted ultrasound survey with a special focus on the orbital region to pregnant women who have children with congenital eye anomalies. Achiron *et al.* [32] reported on axial growth of the fetal eye and the importance of evaluation of the hyaloid artery, its blood flow, and regression.

## Conclusions

These presented cases are examples of rare fetal pathological abnormalities when early diagnosis allowed pregnancy continuation or termination options for parents. Intrauterine diagnosis is not easy or frequent, but ultrasound plays a vital role, complemented by MRI and amniocentesis with chromosome microarray testing. Advances in ultrasound technology, including the use of three-dimensional images, are key to early diagnosis. In both cases, the early diagnosis and recognition of these ocular abnormalities assisted in counseling and support to the patients, and appropriate information could be provided about possible treatment options in neonatal and pediatric life.

A clear diagnosis of microphthalmia was made in Case A. The ultrasound images, however, did not identify thinning in the optic nerve, nor mid-face hypoplasia. MRI is considered to provide a more appropriate imaging modality to identify thinning of the optic nerve, which Patient A declined. In Case B, the right-sided anophthalmia and mild ventriculomegaly was clearly identified on ultrasound. The use of an MRI scan in Patient B was useful to confirm the ultrasound diagnosis and screen for other associated intracranial abnormalities.

The prognosis for fetuses with a prenatal diagnosis of microphthalmia or anophthalmia is complex and frequently uncertain. We recommend chromosomal microarray testing either as an antenatal invasive testing or on a fetal cord sample after delivery. This will provide useful information on possible genetic-associated conditions, and informs future family planning. When a genetic cause is identified, it is prudent to offer formal genetic counseling and parental testing. The use of MRI to image possible anomalies in the fetal brain provides a further indication of whether significant mental impairment can be expected. However, none of these investigations can be undertaken without first diagnosing the condition. The current guidelines for imaging the fetal face, as discussed above, vary from no recommendations for imaging of the orbits, to a simple recommendation to image the orbits, with only the Fetal Medicine Foundation guidelines recommending that internal structures of the eyes are imaged [29].

The sophistication of the latest generation of ultrasound machines and software, combined with the seriousness of a diagnosis of microphthalmia or anophthalmia, are such that it is time to include a robust, consistent protocol for the detailed examination of intraocular structures. We suggest using a curvilinear transducer (2–9 MHz) and imaging orbits in both axial and coronal planes. This can be aided by using a transvaginal probe in selected cases if it is difficult to visualize structures via the transabdominal method. Three-dimensional reverse-face imaging should be obtained in suspected cases of anophthalmia or microphthalmia. The presence or non-visualization of lenses and presence or absence of hyaloid arteries should be documented in reports so that cases can then be appropriately referred for a tertiary ultrasound scan and fetal medicine review.

## Abbreviations

MRI: Magnetic resonance imaging
SNP: Single nucleotide polymorphisms
TORCH: Toxoplasmosis, rubella, cytomegalovirus, herpes simplex virus, listeria, parvovirus, and HIV
